# Assessing Choroidal Neovascular Membrane Flow Regression Using Optical Coherence Tomography Angiography After a Single Injection of Faricimab in Age-Related Macular Degeneration: A Case Study

**DOI:** 10.7759/cureus.73760

**Published:** 2024-11-15

**Authors:** Ahmed B Alsatrawi

**Affiliations:** 1 Ophthalmology, Government Hospitals, Manama, BHR

**Keywords:** age-related macular degeneration (armd), amd, anti-vegf therapy, choroidal neovascularization, cnvm, octa, optical coherence tomography angiography, retina

## Abstract

This case study aims to evaluate the regression of the vascular network in a patient with age-related macular degeneration (AMD) following a single injection of faricimab, utilizing optical coherence tomography angiography (OCTA) for detailed assessment. An elderly patient diagnosed with neovascular AMD received a single injection of faricimab, with pre and post-treatment OCTA imaging performed to assess changes in the choroidal neovascular membrane (CNVm) and surrounding retinal structures. OCTA revealed significant regression of the vascular network following the single injection of faricimab, demonstrating a marked reduction in blood flow within the CNV, which corresponded with structural and anatomical improvements in the retina. This case highlights the potential of faricimab to induce rapid regression of CNVm in AMD after a single injection, as observed through OCTA. Furthermore, while current monitoring primarily relies on OCT B-scan structural assessments, the flow dynamics captured by OCTA may play a crucial role in future treatment monitoring and therapeutic strategies for managing neovascular AMD.

## Introduction

The advancement of optical coherence tomography (OCT) has revolutionized the field of ophthalmology, particularly in the assessment of retinal diseases. OCT allows for high-resolution imaging of the retinal layers, enabling clinicians to visualize structural changes associated with various pathologies, including choroidal neovascular membrane (CNVm) related to age-related macular degeneration (AMD) [1]. The introduction of OCT angiography (OCTA) further enhanced these capabilities by providing non-invasive visualization of blood flow in the retina and choroid, allowing for the detailed examination of neovascularization without the need for dye injection, as required in traditional fluorescein angiography (FFA) [2].

OCTA has proven particularly valuable in assessing the outer retina layers, where CNVm is often localized. This technique facilitates the identification of flow changes and the characterization of neovascular networks, leading to more precise diagnoses and treatment plans [3]. The increased reliance on OCTA has contributed to a significant reduction in the use of FFA, minimizing patient exposure to the risks associated with dye injections and improving patient comfort [4-6].

In the management of CNVm associated with AMD, anti-vascular endothelial growth factor (anti-VEGF) therapies have become the cornerstone of treatment. These agents target the underlying pathophysiology of CNVm by inhibiting VEGF, a key mediator of neovascularization [7]. Traditional anti-VEGF therapies, such as ranibizumab and aflibercept, have demonstrated efficacy in improving visual acuity and reducing fluid accumulation [8]. Recently, the introduction of faricimab, a bi-specific antibody that simultaneously inhibits both VEGF-A and angiopoietin-2, has opened new avenues for treatment. Faricimab's dual mechanism of action may enhance its therapeutic effectiveness in managing CNVm related to AMD, as evidenced by promising clinical trial results [9].

This case report discusses a patient with AMD and active CNVm who experienced complete regression of neovascularization following the first injection of faricimab. This highlights the early and significant effects of this medication on the vascular network in the outer retina slab. Additionally, this case underscores that anatomical success precedes functional success, as the patient’s vision improved after the third injection.

## Case presentation

A 59-year-old female with no significant medical history presented in July 2024 with a one-month history of decreased vision in her left eye. Upon clinical examination, her right eye demonstrated a visual acuity of 6/6, with a normal slit lamp exam revealing only drusen in the macular region. In contrast, the left eye had a visual acuity of 6/24, and the clinical exam revealed the presence of drusen, CNVm, nearby retinal hemorrhage, and significant macular edema.

**Figure 1:**
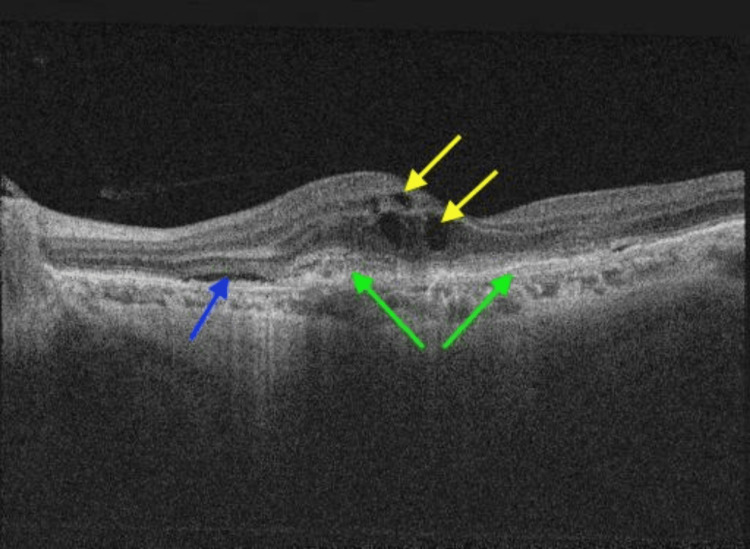
Radial OCT B-scan (12 mm) of the left eye macula at presentation Active CNVm in the left eye macula is indicated by the presence of sub-RPE hyper-reflective material (green arrows). Associated findings include sub-retinal fluid (blue arrow) and intra-retinal fluid (yellow arrows), which reflect the ongoing activity of the neovascularization. OCT: optical coherence tomography; CNVm: choroidal neovascular membrane; RPE: retinal pigment epithelium

**Figure 2:**
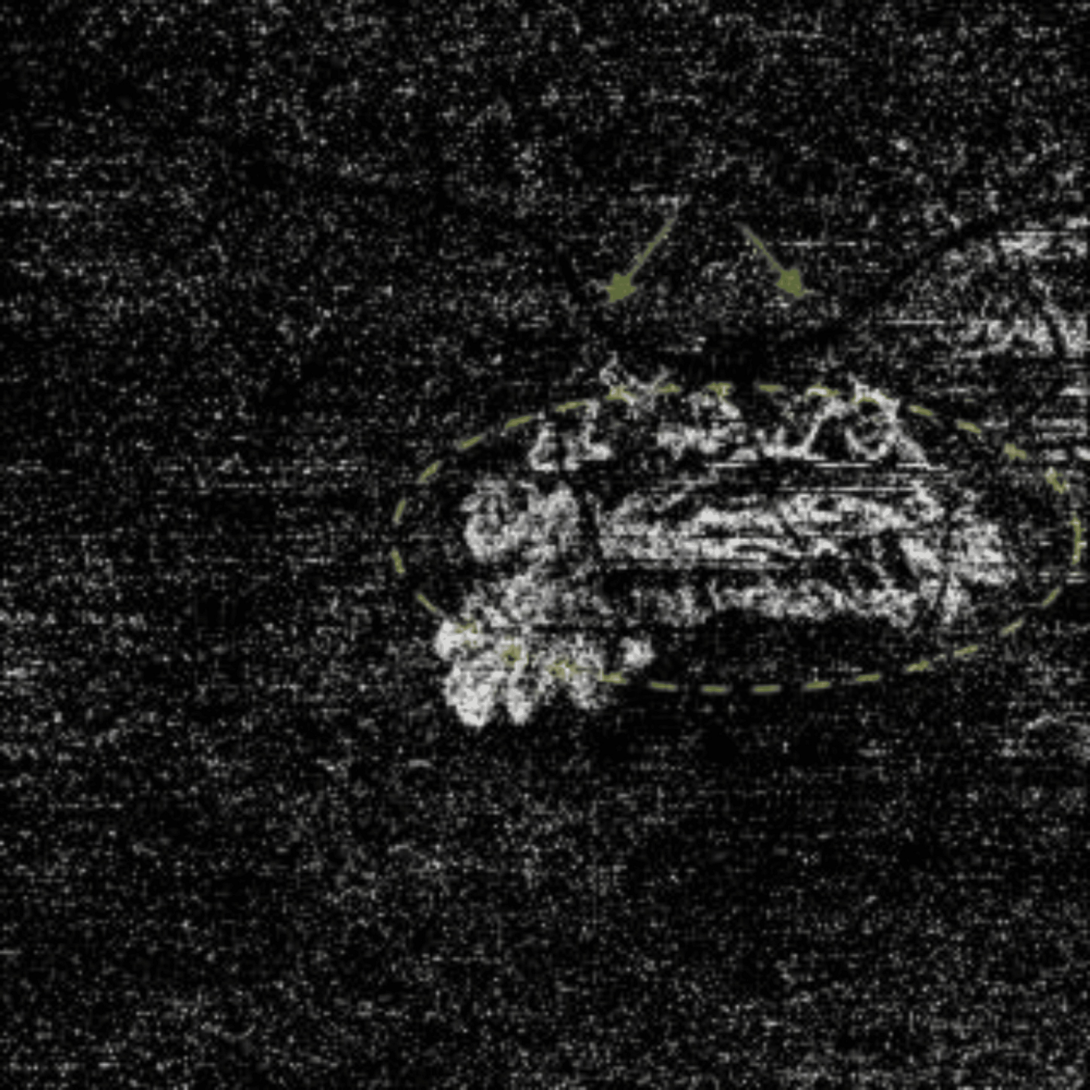
En face OCTA of the outer retina slab (4.5 mm X 4.5 mm) at presentation The en-face scan of the OCTA image of the outer retina slab exhibits a predominantly dark black appearance with some granularity, indicating the absence of blood flow in this layer. The active CNVm flow network is highlighted by a yellow dashed oval, clearly delineating the are of vascular activity. The projection artifact of the retina vessel (green arrow) is used as a location reference. OCTA: optical coherence tomography angiography; CNVm: choroidal neovascular membrane

**Figure 3:**
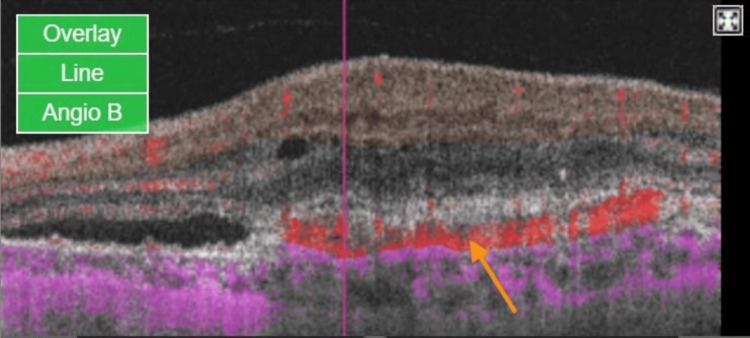
OCT B-scan with flow (Angio B) at the center of the CNVm at presentation The OCT B-scan is overlaid with color-coded flow information, where red indicates blood flow within the retina and purple represents flow in the choroid. The marked red flow within the CNVm (orange arrow) emphasizes active vascularization in the context of the underlying retina. OCTA: optical coherence tomography angiography; CNVm: choroidal neovascular membrane

Both OCT and OCTA were performed using a swept-source Topcon Triton imaging system. The OCT B-scan demonstrated intra-retinal fluid and sub-retinal fluid, along with a hyper-reflective membrane beneath the retinal pigment epithelium (RPE), suggestive of macular neovascularization (MNV) type 1 (Figure 1). The en-face OCTA slab of the outer retina revealed a network of vessels with positive flow (Figure 2), correlating with the same sub-RPE hyper-reflective area of the MNV identified on the OCT B-scan, further confirmed by positive flow on Angio B (Figure 3).

**Figure 4:**
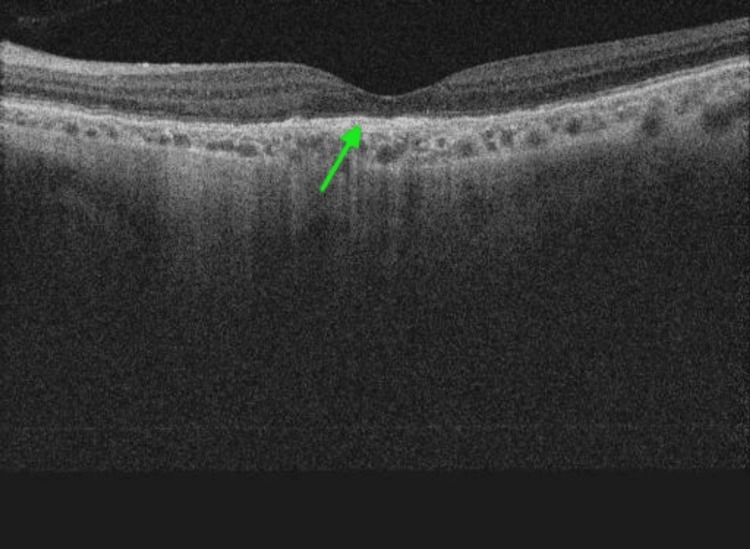
Radial OCT B-scan (12 mm) of the left eye macula one month after the first injection The OCT B-scan demonstrates the absence of both sub-retinal and intra-retina fluid, indicating the inactivity of the CNVm. It is identified as sub-RPE hyper-reflective material (green arrow). OCT: optical coherence tomography; CNVm: choroidal neovascular membrane; RPE: retinal pigment epithelium

**Figure 5:**
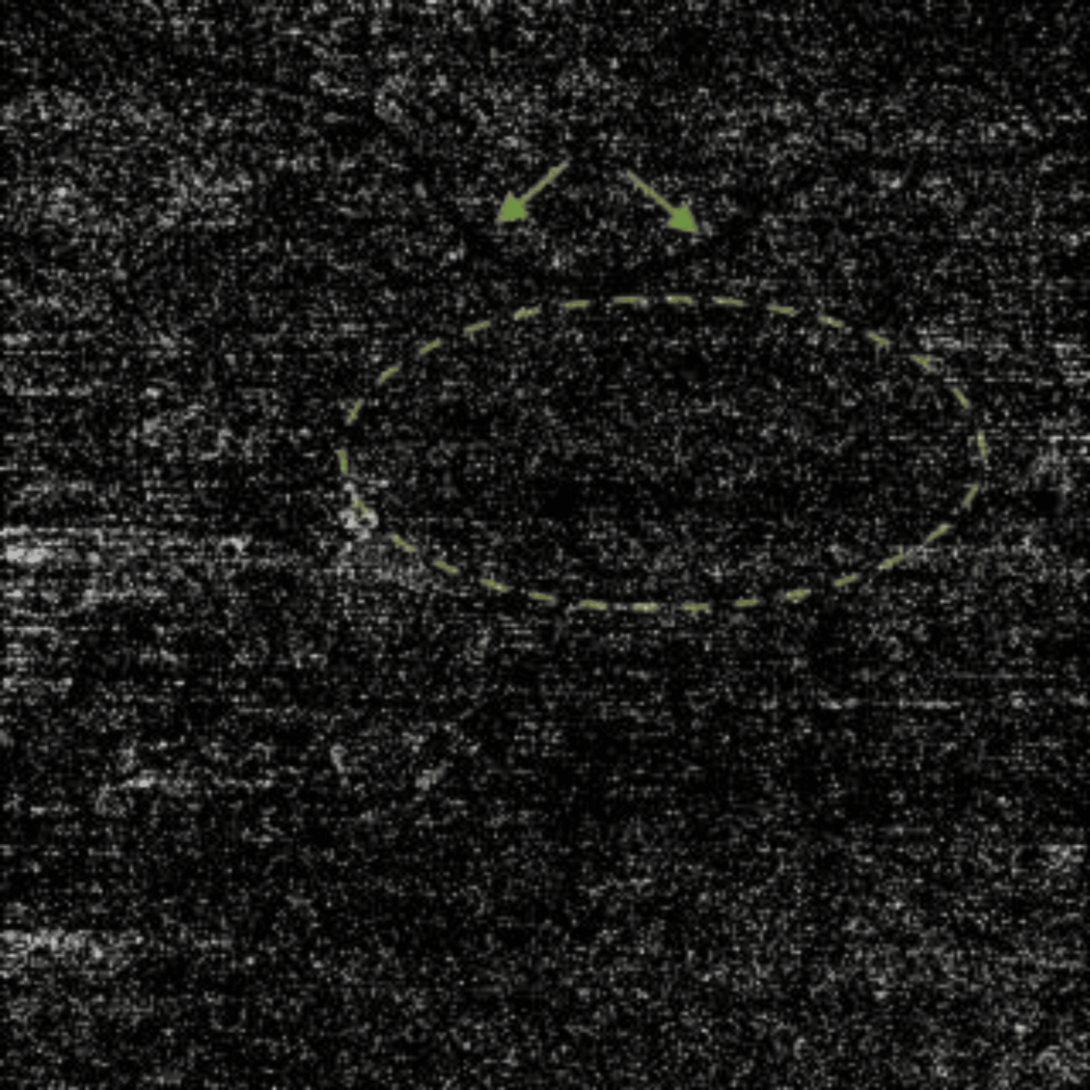
En face OCTA of the outer retina slab (4.5 mm X 4.5 mm) one month after the first injection En face OCTA of the outer retina slab appears entirely black, indicating an absence of blood flow in the entire frame. The yellow oval outlines an area where no vascular network is present, further confirming the lack of detectable flow within the CNVm, the reference retinal artery (green arrows). OCTA: optical coherence tomography angiography; CNVm: choroidal neovascular membrane

**Figure 6:**
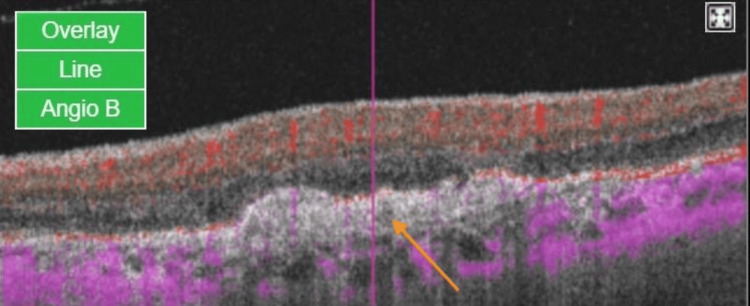
OCT B-scan with flow (Angio B) at the center of the CNVm one month after the first injection In this OCT B-scan overlaid with color-coded flow information, the absence of flow in the CNVm is noted by the disappearance of the red color seen earlier at the presentation. No flow signals are seen at the CNVm (orange arrow). OCT: optical coherence tomography; CNVm: choroidal neovascular membrane

The patient was diagnosed with age-related macular degeneration (AMD) and active CNVm and was initiated on a loading course of four faricimab injections. During her follow-up visit one month later, before her second injection, her visual acuity in the left eye remained at 6/24. However, the OCT B-scan revealed a complete resolution of fluid, indicating a dry macula (Figure 4). Importantly, OCTA showed complete regression of the vascular network in the outer retina slab (Figure 5), with no detectable flow on the Angio B OCT (Figure 6). The OCTA was repeated three times, and manual segmentation was performed to confirm the absence of flow. This anatomical success persisted following the third loading injection; at which point, the patient experienced a functional improvement in visual acuity, reaching 6/12.

## Discussion

The regression of the vascular network in the outer retina slab after anti-VEGF treatment in patients with AMD has been documented in several studies. Notably, complete regression of CNVm after a single injection of anti-VEGF agents has been reported, although such outcomes are often variable and dependent on individual patient factors. Many studies have observed that while some patients may show rapid and complete regression, others may require multiple injections to achieve similar results (10-15).

The significance of observed regression in OCTA is multifaceted. A decrease in flow may indicate either a reduction in blood flow speed below the detection threshold of the imaging system or a more profound closure of the neovascular network [10-12]. Studies have suggested that while flow cessation may occur, the anatomical closure of CNVm can take longer to achieve, leading to the hypothesis that some networks may still be present but not detectable due to low flow [11-15].

Faricimab, a bi-specific antibody targeting both VEGF-A and angiopoietin-2, has shown promising efficacy in the treatment of CNVm associated with AMD. Clinical trials have indicated that faricimab can lead to significant improvements in visual acuity and anatomical outcomes [16]. However, there has been a debate among retina specialists regarding the timing of its effects compared to older and more commonly used therapies like aflibercept and ranibizumab. Some clinicians have reported a perceived delay in the efficacy of faricimab, suggesting that it may not produce immediate results as rapidly as its predecessors. This case report challenges that notion by demonstrating a complete regression of the vascular network after the first injection.

In the existing literature, there have been limited reports detailing complete regression of CNVm specifically after the first injection of Faricimab. A thorough review of recent studies indicates that while faricimab has demonstrated significant efficacy in treating CNVm, documented cases of immediate complete regression following a single injection remain sparse [16-18]. Thus, this case may represent one of the earliest reports of such a complete regression after the first faricimab injection in the context of AMD.

Additionally, recent literature has begun to correlate OCTA findings with treatment outcomes in patients receiving faricimab. Studies have indicated that early changes in the vascular network as observed by OCTA may predict long-term treatment responses, suggesting a potential role for OCTA in monitoring therapeutic efficacy and informing treatment decisions [10-13].

In patients receiving anti-VEGF treatment for AMD, anatomical success does not always translate quickly into functional improvement. In this case, while complete anatomical regression was observed after the first injection, it took three injections for the patient to experience a notable improvement in visual acuity. This highlights the complexity of correlating anatomical changes to functional outcomes, as the relationship between retinal anatomy and vision remains unclear and is still under investigation [19-20].

## Conclusions

While regression of CNVm after a single injection of anti-VEGF agents can occur, significant variability exists among patients. Understanding the implications of this regression, whether it indicates complete closure of the neovascularization or merely a reduction in flow, requires further investigation. This case provides valuable insights into the efficacy of faricimab, particularly in relation to OCTA findings, which reveal important anatomical changes. However, it is essential to recognize the limitations inherent to case reports, including the variability of individual responses to treatment, which may not be representative of broader patient populations. The generalizability of these findings is limited, emphasizing the need for larger, controlled studies to validate the efficacy observed in this case. Additionally, the observed delay in functional improvement following anatomical success underscores the complexity of managing age-related macular degeneration (AMD). These insights highlight the necessity for ongoing research to better understand the relationship between vascular changes and visual outcomes, ultimately enhancing treatment strategies for patients with AMD.
